# Plasma biomarker profiles in ageing: decoding neurodegeneration *in vivo*

**DOI:** 10.1093/brain/awaf281

**Published:** 2025-08-07

**Authors:** Niklas Mattsson-Carlgren

**Affiliations:** Clinical Memory Research Unit, Department of Clinical Sciences Malmö, Faculty of Medicine, Lund University, 202 13 Malmö, Sweden; Memory Clinic, Skåne University Hospital, 205 02 Malmö, Sweden; Wallenberg Center for Molecular Medicine, Lund University, 221 84 Lund, Sweden

## Abstract

This scientific commentary refers to ‘Neuropathologic correlates of distinct plasma biomarker profiles in community-living older adults’ by Yu *et al*. (https://doi.org/10.1093/brain/awaf211).

**This scientific commentary refers to ‘Neuropathologic correlates of distinct plasma biomarker profiles in community-living older adults’ by Yu *et al*. (https://doi.org/10.1093/brain/awaf211).**

Plasma biomarkers have emerged as powerful tools for mapping different pathological processes *in vivo* in patients with neurodegenerative diseases. The success of this approach has been especially striking in Alzheimer’s disease (AD), where plasma biomarkers now rival the diagnostic accuracy of specialized investigations such as CSF analyses or PET scans.^1^ Recently, plasma biomarkers have even received FDA approval for clinical use.^2^ With their unprecedented scalability, plasma biomarkers have the potential to revolutionize personalized medicine for patients with cognitive impairments.

Despite this progress, a number of gaps remain in our understanding of plasma biomarkers for neurodegenerative diseases. Most studies to date have lacked post-mortem confirmation of pathology, which is essential to fully appreciate the complex patterns of co-existing pathologies in the ageing brain, and their effects on plasma biomarker levels. Studies are therefore needed that integrate promising plasma biomarkers with detailed neuropathological data to explore how different plasma biomarkers may be used together to elucidate patterns of brain pathology.

The new study by Yu and co-workers^3^ in this issue of *Brain* represents a significant contribution to this area, by offering a data-driven framework for interpreting the discrete and overlapping presence of neurodegenerative processes using different plasma biomarkers. The study included 405 participants from three neuropathology cohorts, with participants spanning the full continuum from cognitively normal to dementia. The participants, who had an average age of ∼89 years at death, were richly phenotyped both with neuropathological data (including nine different features such as distinct proteinopathies and vascular brain changes) and plasma biomarker data (including amyloid-β_42/40_, p-tau217, NFL and GFAP). Plasma samples were collected on average 4 years before death.

Most of the participants had some degree of neuropathology, and mixed pathologies were common, with nearly 80% having at least two different neuropathological changes. The most common were AD, cerebral amyloid angiopathy (CAA), infarcts, limbic-predominant age-related TDP-43 encephalopathy (LATE) and Lewy bodies. Overlap between AD and CAA and/or LATE was especially common. It is worth noting that the cohort studied here was community-based rather than clinic-based. One consequence of this is a low prevalence of atypical or rare neuropathologies [e.g. frontotemporal lobar degeneration (FTLD)], which typically have higher prevalences in more specialized settings.^4^

The authors tested associations both for individual biomarkers and for patterns of biomarkers, using latent profile analysis. Evaluation of different cluster solutions supported a system with three biomarker profiles, which the authors then compared with neuropathological features (Fig. 1). The most common profile (Profile 1, ∼56%) was characterized by comparatively low plasma levels of p-tau217, NFL and GFAP, and high levels of amyloid-β (Aβ)_42/40_. (Note that this study did not report positivity or negativity of biomarkers but compared continuous levels of biomarkers between profiles.) This biomarker profile was the most common among cognitively normal participants, although it was also seen in patients with mild cognitive impairment or dementia. Despite the relatively more normal biomarker levels (in comparison to the other identified profiles), ∼51% of individuals with Profile 1 exhibited intermediate AD neuropathology, and other pathologies were also quite common, indicating that this profile was not strictly associated with an absence of pathological changes.

**Figure 1:**
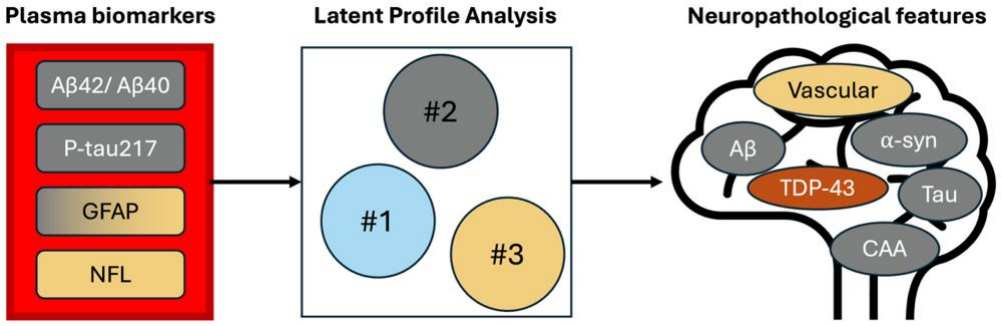
**Linking plasma biomarkers to neuropathological features.** Four plasma biomarkers were assessed and used in a data-driven fashion to define three groups of participants. These groups were next characterized according to their expression of different neuropathologies. Profile 2 was enriched for Alzheimer’s disease (AD), cerebral amyloid angiopathy (CAA) and α-synuclein-related features, while Profile 3 was enriched for vascular features. There was not a complete overlap between biomarker profiles and neuropathological features, and some neuropathologies did not differ at all between biomarker profiles. Aβ = amyloid-β.

The second most common profile (Profile 2, ∼35%) was most often seen in patients with mild cognitive impairment or dementia and was characterized by elevated p-tau217 and GFAP levels, and a high prevalence of AD neuropathology. The dominance of p-tau217 here mirrors its role as a central biomarker of amyloid pathology in AD, as reported in other studies.^5^ P-tau217 was associated with both amyloid and tau pathologies, while elevated GFAP levels appeared most strongly associated with tau tangle density. Profile 2 also showed relatively high prevalences of Lewy bodies and CAA, consistent with previous reports suggesting possible mechanistic links between build-up of amyloid, tau and α-synuclein pathology.^6^

A remaining challenge for the field is to disentangle patients with AD with and without co-occurring α-synuclein pathology *in vivo* (which may have relevance both in clinical practice and in clinical trials). Biomarkers in CSF, based on seeding amplification assays,^7^ show promising results for detection of α-synuclein pathology *in vivo*, but such biomarkers remain to be developed and validated for blood or other tissues where sampling is more readily available than CSF.^8^

The third and least common profile (Profile 3, ∼10%) was primarily seen in patients with dementia, and was characterized by increases in NFL and GFAP levels and a high burden of various vascular pathologies. Previous studies have found increased plasma NFL across a range of neurodegenerative conditions, establishing it as a relatively non-specific measure of neuronal injury.^9^ Similarly, high GFAP levels across Profiles 2 and 3 may reflect astrocytic involvement in both AD-related and vascular-related injury.

Taken together, the findings in this study add to evidence that plasma biomarkers can serve as *in vivo* measures of specific brain pathologies. The study also highlights important remaining gaps, such as plasma biomarkers to identify TDP-43 (LATE) or α-synuclein (Lewy body) pathologies, two common neuropathological features that were not associated with any of the biomarkers tested here. The study has many strengths, including a large sample size, a community-based cohort, a detailed neuropathological characterization, and an unbiased data-driven approach to exploring biomarker patterns.

However, while this was a rich analysis, the average lag of 4 years between plasma sampling and death complicates the interpretation of findings. Some of the pathological changes may have developed during the interval between sampling and death or increased in severity in a way that would alter their associations with biomarkers. For example, it is possible that some of the AD biomarkers (e.g. plasma p-tau217) would have been able to differentiate more clearly between presence or absence of AD pathology if sampled closer to death. A shorter lag between plasma sampling and death might also have resulted in more fine-grained and less heterogenous profile groups.

In addition, the participants had a high average age, and it is not certain the results would fully generalize to younger populations. In fact, the advanced age likely contributed to the high prevalence of co-pathologies, which in turn complicates the disentanglement of biomarker relationships. The overlap between plasma biomarker profiles remained considerable for several of the neuropathological features. Nonetheless, these cohorts remain an invaluable resource that could benefit from further analyses with even more recently developed biomarkers, e.g. plasma eMTBR-tau243 for AD-related aggregated tau pathology.^10^ Broader -omics studies (e.g. proteomics) may also uncover more nuanced biochemical changes associated with specific neuropathological features.

To conclude, Yu and co-workers^3^ provide a fascinating view into clinically relevant biomarker patterns, which may help clinicians and researchers to interpret plasma biomarkers in the context of the complex co-pathologies that characterize the ageing brain.
